# The Role of Abscisic Acid (ABA) Machinery in Stress Response

**DOI:** 10.3390/plants14060935

**Published:** 2025-03-17

**Authors:** Gastón A. Pizzio

**Affiliations:** 1 Institute for Integrative Systems Biology (I2SysBio), Universitat de València-CSIC, 46908 Paterna, Spain; gapizzio@gmail.com; 2 CIT-RIO NEGRO, Universidad Nacional de Río Negro, 8500 Viedma, Argentina

## 1. Introduction

Increasing global temperatures, in tandem with predicted increases in future frequencies of drought and flooding episodes, represent a threat to agricultural productivity. They necessitate the incorporation of mitigation strategies and, consequently, the need to address crop water use efficiency, ensuring food security.

The phytohormone abscisic acid (ABA) is a key regulator in plant physiology, playing important roles in drought, heat, and salinity tolerance, among other abiotic stresses [1,2,3]. ABA is also both a growth regulator under normal conditions and a developmental cue. Its machinery includes components ranging from synthesis to molecular perception, signaling, and response, which are currently being studied in terms of fine regulation.

This Special Issue contains six research articles that focus on ABA signaling and response in the context of plant abiotic stress, from model species (arabidopsis and setaria) to crops such as *Vitis vinifera* and *Camellia oleifera*. These articles can improve understanding of how ABA machinery works at a basic level, providing an advantage in overcoming the negative effects of climate change in food productivity.

## 2. Overview of This Special Issue’s Articles

**Figure 1:**
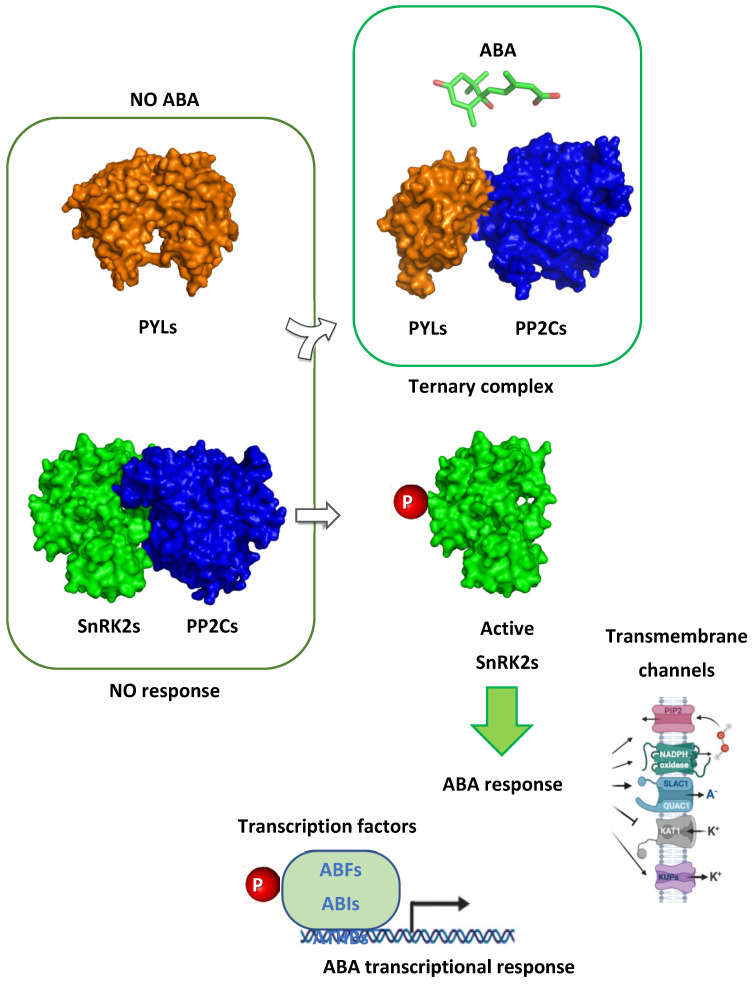
ABA signaling and response. ABA is perceived by PYR/PYL/RCAR receptors, followed by ternary complex establishment with clade A PP2Cs. In turn, the formation of this ternary complex leads to SnRK2 release and activation, triggering ABA response through ABA-induced target phosphorylation at different subcellular levels.

Abiotic stresses, such as drought, heat, and salinity, have a strong negative impact on plant development and reproduction, altering flowering patterns and reducing biomass and seed number. In plants, ABA regulates stress responses through a signaling pathway composed of positive (receptors and kinases) and negative elements (phosphatases). Upon PYR/PYL/RCAR receptors perceiving ABA, a ternary complex with clade A PP2C phosphatases is established (Figure 1). In this situation, SnRK2 kinases are released by the inhibition of PP2C phosphatases and then activated via reversible phosphorylation, and a myriad of effectors are targeted, from transmembrane proteins to transcription factors [4]. As a result, different abiotic stress tolerance mechanisms are triggered, such as stomatal closure, antioxidant machinery activation, and osmocompatible solute production [5].

In this context, Peres de Oliveira et al. [6] explored the role of ABA core signaling components on drought tolerance induction in both *Setaria italica* and *Setaria viridi.* They found that ABA core signaling components, such as PYR/PYL/RCARs, PP2Cs, and SnRK2s, are highly conserved between Setaria spp. Moreover, drought stress response triggers negative feedback for ABA core signaling gene expression, downregulating PYL genes (a positive component of the ABA signaling pathway) and upregulating PP2C genes (a negative component). These findings demonstrate key genes that have great potential as targets for drought resistance induction and crop breeding strategies.

Huang et al. [7] evaluated the negative role of the Arabidopsis protein phosphatase PIA1 on the ABA signaling pathway. PIA1 is a PP2C clade F member, and its gene expression is downregulated after ABA treatment; its overexpression is also able to downregulate ABA-induced gene expression. In fact, PIA1ox lines were less sensitive to ABA in seed germination, root growth, and stomatal opening assays, whereas pia1 mutant lines showed the opposite trait. Moreover, under drought stress, the antioxidant capacity and survival rate of PIA1ox lines were reduced with respect to Col-0 and pia1 mutant lines. Additionally, PIA1 protein was found to interact with ABA core signaling positive components, such as RCARs and SnRK2s. Overall, PIA1 may reduce plant drought tolerance by functioning as a common negative regulator of the ABA signaling pathway.

Murcia et al. [8] studied the impact of ABA and GA3 on the antioxidant capacity of Malbec grapevine berries during ripening. Using quantitative proteomics, they revealed that ABA and GA3 treatment enhanced antioxidant defense protein expression, inducing H_2_O_2_ scavenging. Additionally, they found increased levels of the stilbenes, such as E-viniferin and quercetin, in the treated berries, which also enhanced H_2_O_2_ scavenging and sugar contents at the ripe berry stage. This research provides important insights in the role of ABA and GA3 in wine cultivar grape quality, which is highly beneficial for viticulture and wine production.

Yang et al. [9] characterized ABA-induced drought stress tolerance in *Camellia oleifera* at the physiological and biochemical levels. They showed that foliar ABA spray application regulates stomatal conductance, photosynthesis, oxidative stress response, and osmotic balance, leading to drought stress tolerance. Of note, ABA treatment effectively activated the antioxidant system, alleviating oxidative damage caused by drought stress while also promoting the synthesis of osmotic regulators such as proline, maintaining cellular turgor stability. This work increases the understanding of drought stress response in *C. oleifera* and provides a starting point for the development of drought resistance management strategies.

Since a large portion of ABA response genes remain undiscovered, Li et al. [10] studied the regulation of ABA response by DUF38 proteins in Arabidopsis. They identified a subgroup of DUF538 proteins called ASDs (ABA-inducible signal peptide-containing DUF538 proteins), which regulate plant responses to ABA. The results showed that the expression levels of ASDs increased significantly in response to ABA, NaCl, and mannitol treatments. However, genetic and physiological characterization showed that ASD1/ASD3 and ASD2/ASD4 have opposite functions. For instance, seed germination and cotyledon greening assays showed an ABA sensitivity increment in lines that overexpressed ASD1 or ASD3, but a decrease in plants that overexpressed ASD2 or ASD4. In agreement, ABA sensitivity was increased in asd2 CRISPR/Cas9 gene-edited lines but reduced in the asd3 single mutants. On the other hand, through differential gene expression analysis, they showed that ASD2 is involved in different processes, such as plant growth and development, secondary metabolism, and plant hormone signaling. This work improves our knowledge about the fine-tuning of ABA transcriptomic response, and can be used to create crop breeding plans to cope with the challenges imposed by climate change.

Hussain et al. [11] studied the role of AtbZIP62 transcription factor on ABA transcriptomic response in Arabidopsis. They found that ABA induced AtbZIP62 expression and demonstrated AtbZIP62 nuclear subcellular localization and its function as a transcriptional repressor. Moreover, 35S:AtbZIP62 transgenic lines were hypersensitive to ABA-regulated seed germination and cotyledon greening assays, while atbzip62 mutant lines were hyposensitive to ABA. Additionally, transcriptome analysis with differentially expressed genes (DEGs) showed enrichment in processes such as response to abiotic stresses and response to ABA, proving that AtbZIP62 is an ABA-responsive gene with transcriptional repressor activity that in turn positively regulates ABA transcriptional responses.

## 3. Conclusions

The articles in this Special Issue shed light on important mechanisms related to ABA signaling and abiotic stress tolerance. As Guest Editor, I am grateful to all authors who contributed such high-quality research. I am convinced that this Special Issue can provide valuable knowledge for further improving the development of agronomical strategies in order to cope with crop productivity in harsh environments.

## Data Availability

The information discussed in this editorial note are available on-line (see references).
